# Simple motion correction strategy reduces respiratory-induced motion artifacts for k-t accelerated and compressed-sensing cardiovascular magnetic resonance perfusion imaging

**DOI:** 10.1186/s12968-018-0427-1

**Published:** 2018-02-01

**Authors:** Ruixi Zhou, Wei Huang, Yang Yang, Xiao Chen, Daniel S. Weller, Christopher M. Kramer, Sebastian Kozerke, Michael Salerno

**Affiliations:** 1 Departments of Medicine, Cardiovascular Imaging Center, University of Virginia Health System, Charlottesville, VA USA; 2 Department of Biomedical Engineering, Cardiovascular Imaging Center, University of Virginia Health System, Charlottesville, VA USA; 3 Medical Imaging Technologies, Siemens Healthineers, Princeton, NJ USA; 4 Department of Electrical and Computer Engineering, University of Virginia, Charlottesville, VA USA; 5 Department of Radiology, Cardiovascular Imaging Center, University of Virginia Health System, Charlottesville, VA USA; 6 Department of Information Technology and Electrical Engineering, Institute for Biomedical Engineering, University and ETH Zurich, Zurich, Switzerland; 7 Cardiovascular Division, University of Virginia Health System, 1215 Lee Street, PO Box 800158, Charlottesville, VA 22908 USA

**Keywords:** CMR perfusion, Motion compensation, K-t acceleration, Image reconstruction, Myocardial perfusion

## Abstract

**Background:**

Cardiovascular magnetic resonance (CMR) stress perfusion imaging provides important diagnostic and prognostic information in coronary artery disease (CAD). Current clinical sequences have limited temporal and/or spatial resolution, and incomplete heart coverage. Techniques such as k-t principal component analysis (PCA) or k-t sparcity and low rank structure (SLR), which rely on the high degree of spatiotemporal correlation in first-pass perfusion data, can significantly accelerate image acquisition mitigating these problems. However, in the presence of respiratory motion, these techniques can suffer from significant degradation of image quality. A number of techniques based on non-rigid registration have been developed. However, to first approximation, breathing motion predominantly results in rigid motion of the heart. To this end, a simple robust motion correction strategy is proposed for k-t accelerated and compressed sensing (CS) perfusion imaging.

**Methods:**

A simple respiratory motion compensation (MC) strategy for k-t accelerated and compressed-sensing CMR perfusion imaging to selectively correct respiratory motion of the heart was implemented based on linear k-space phase shifts derived from rigid motion registration of a region-of-interest (ROI) encompassing the heart. A variable density Poisson disk acquisition strategy was used to minimize coherent aliasing in the presence of respiratory motion, and images were reconstructed using k-t PCA and k-t SLR with or without motion correction. The strategy was evaluated in a CMR-extended cardiac torso digital (XCAT) phantom and in prospectively acquired first-pass perfusion studies in 12 subjects undergoing clinically ordered CMR studies. Phantom studies were assessed using the Structural Similarity Index (SSIM) and Root Mean Square Error (RMSE). In patient studies, image quality was scored in a blinded fashion by two experienced cardiologists.

**Results:**

In the phantom experiments, images reconstructed with the MC strategy had higher SSIM (*p* < 0.01) and lower RMSE (*p* < 0.01) in the presence of respiratory motion. For patient studies, the MC strategy improved k-t PCA and k-t SLR reconstruction image quality (*p* < 0.01). The performance of k-t SLR without motion correction demonstrated improved image quality as compared to k-t PCA in the setting of respiratory motion (*p* < 0.01), while with motion correction there is a trend of better performance in k-t SLR as compared with motion corrected k-t PCA.

**Conclusions:**

Our simple and robust rigid motion compensation strategy greatly reduces motion artifacts and improves image quality for standard k-t PCA and k-t SLR techniques in setting of respiratory motion due to imperfect breath-holding.

## Background

Adenosine stress first-pass contrast-enhanced cardiovascular magnetic resonance (CMR) perfusion imaging has been shown to have excellent diagnostic and prognostic utility in evaluating coronary artery disease (CAD) [1–4]. It has a number of advantages over other modalities including lack of ionizing radiation, and comparatively higher spatial and temporal resolution.

Clinically available CMR techniques typically have incomplete slice coverage due to temporal and spatial resolution constraints. Current clinical techniques are limited to acquire 3 to 4 short axis slices per heartbeat with a spatial resolution of 2–3 mm and temporal resolution of 100-180 ms [5]. Most clinical techniques use parallel imaging techniques such as sensitivity encoding (SENSE) [6] or generalized autocalibrating partially parallel acquisition (GRAPPA) [7] at low acceleration factors of 2 to 3. These techniques typically fully sample a central region of k-space for auto-calibration of coil sensitivity or GRAPPA kernels which slightly reduces the effective acceleration rate. Alternatively, techniques such as temporal SENSE (TSENSE) [8] and temporal GRAPPA (TGRAPPA) [9] can be used to reduce the temporal footprint by utilizing time averaged data for kernel calibration or sensitivity maps in lieu of acquiring a fully-sampled k-space center during each heartbeat. A number of spatial-temporal accelerated techniques such as k-t broad-use linear acquisition speed-up technique (BLAST)/k-t sensitivity encoding (SENSE) [10] and k-t principal component analysis (PCA) [11] have been developed to address these limitations and have demonstrated excellent clinical performance [12–14]. These techniques rely on the high degree of spatiotemporal correlation within the dynamic dataset to constrain the image reconstruction. Recently, compressed sensing (CS) techniques [15] have been applied to myocardial perfusion imaging [16–19]. These techniques use a non-linear reconstruction to recover the images from incoherent under-sampled data; relying on the fact that the dynamic image data has a sparse representation in some transform domain.

Although the above fast imaging techniques can achieve high acceleration rates, in the presence of respiratory motion, these techniques can suffer from significant degradation of image quality [20, 21]. Robust routine clinical application of these techniques can be limited by their sensitivity to respiratory motion-induced artifacts.

A number of approaches for respiratory motion corrected reconstruction have been proposed, but they have limitations. Slice-tracking using navigators [22] has been used to prospectively reduce respiratory motion, but these techniques require a dedicated navigator setup and adequate navigator quality with a clear liver-lung interface.

Further, these techniques are prone to errors resulting from the difference between diaphragmatic motion and cardiac motion. Image-based motion correction strategies [23] use retrospective image registration, in which motion information is extracted from the acquired data and then used to correct data during image reconstruction. In general, the performance of these techniques is highly dependent on the quality of the non-rigid registration, which may be sensitive to changes in signal-to-noise ratio (SNR) and contrast-to-noise ratio (CNR), through-plane motion, or other factors. While multiple methods for non-rigid motion registration have been proposed [24–26], repeated application of non-rigid registration operators can result in image degradation due to spatial interpolation, and can cause geometric distortion of the images. The complexity and potentially long image reconstruction time also limit their general application.

In an electrocardiogram (ECG) gated first-pass myocardial perfusion image sequence, the heart is in the same cardiac phase in each image, and the predominant variation in cardiac position is due to respiratory motion in the head-foot direction. As such, we propose a simple robust respiratory motion compensation strategy for k-t accelerated and compressed-sensing CMR perfusion imaging to selectively correct respiratory motion of the heart. This strategy is based on linear k-space phase shifts derived from rigid motion registration of a heart region of interest (ROI). There are potentially two main advantages for this strategy. First, rigid motion correction tends to be quite robust, and second, the strategy is fast, easy to implement and requires only minor modification to existing reconstruction pipelines.

In terms of sampling strategy, two fundamentally different undersampling strategies have been pursued to recover missing information in dynamic CMR. The first one is uniform undersampling along a sheared grid pattern which has been used for k-t accelerated techniques. Equally spaced rectilinear sampling results in a simple coherent aliasing pattern which is ideal for parallel imaging. However in the setting of respiratory motion, this uniform undersampling results in discrete aliasing artifacts which compromises image quality [20].

While non-uniform Cartesian sampling has been proposed for k-t accelerated techniques such k-t BLAST [10], the non-uniform Cartesian sampling requires iterative reconstruction techniques [27]. For CS techniques, non-uniform undersampling is a prerequisite to satisfy the incoherent sampling criteria of compressed sensing.

As first-pass perfusion images are typically ECG triggered, the heart remains in a relatively similar cardiac phase and respiratory position, and hence the meaningful dynamic information is the change in image intensity due to contrast passage. We hypothesize that a combination of incoherent sampling and a simple rigid motion correction of the whole image based on respiratory motion estimates, derived from a ROI around the heart, would substantially improve the image quality for k-t accelerated and temporal CS perfusion imaging, particularly in the setting of poor breath-holding.

In this work, we propose and test a simple respiratory motion compensation strategy for k-t accelerated and compressed-sensing CMR perfusion imaging to selectively correct respiratory motion of the heart, based on linear k-space phase shifts derived from rigid motion registration of a heart region of interest.

## Methods

### Motion correction strategy

**Fig. 1:**
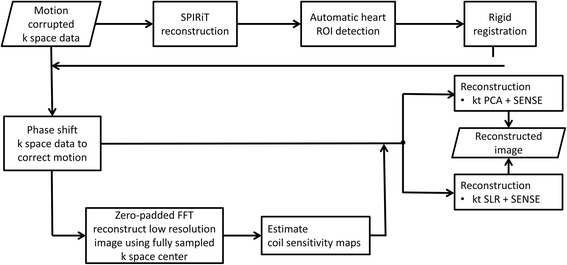
Reconstruction pipeline

The reconstruction pipeline utilized for our motion-compensated reconstruction strategy is depicted in Fig. 1. First, the data from each frame are independently reconstructed using self-consistent parallel imaging reconstruction SPIRiT [28] to obtain images of sufficient quality for rigid registration. This parallel-imaging only approach is used to derive the motion estimates without the temporal blurring that occurs with k-t or CS acceleration in the setting of respiratory motion. Next a 40 × 40 pixel ROI containing the heart is automatically detected based on the fact that the ventricular cavities have the largest magnitude of change in signal intensity over the first-pass of the contrast. Thus, the ROI containing the heart can be automatically detected by finding the largest connected region of high standard deviation on a standard deviation map of signal intensity calculated from all of the frames of the dynamic dataset. Next, rigid registration is performed over the square heart ROI to determine the in-plane displacements required to compensate for the bulk changes in the heart position resulting from respiratory motion. While breathing results in non-rigid motion of structures of the chest, the motion of a small rectangular ROI around the heart on an ECG gated short-axis image can be reasonably approximated by in-plane rigid motion in the head-foot and anterior-posterior directions. Rigid registration was performed by using mutual information as a metric to determine the rigid transformation from the source image to that of the target image [29]. In order to minimize respiratory drift, pairwise rigid registration of images was performed over a 15-frame window, which means the n^th^ frame is registered from (n-7)^th^ to (n + 7)^th^ frame. The obtained displacement information is used to derive the appropriate k-space linear phase shifts to register the heart throughout the temporal series. These linear phase shifts are then applied to the acquired raw k-space data for each frame of the dynamic dataset. Note that this will selectively register the region around the heart and potentially result in suboptimal registration of the structures remote from the heart. This registered raw data is then reconstructed using a conventional k-t PCA or k-t SLR reconstruction strategy.

### Data sampling strategy

**Fig. 2:**
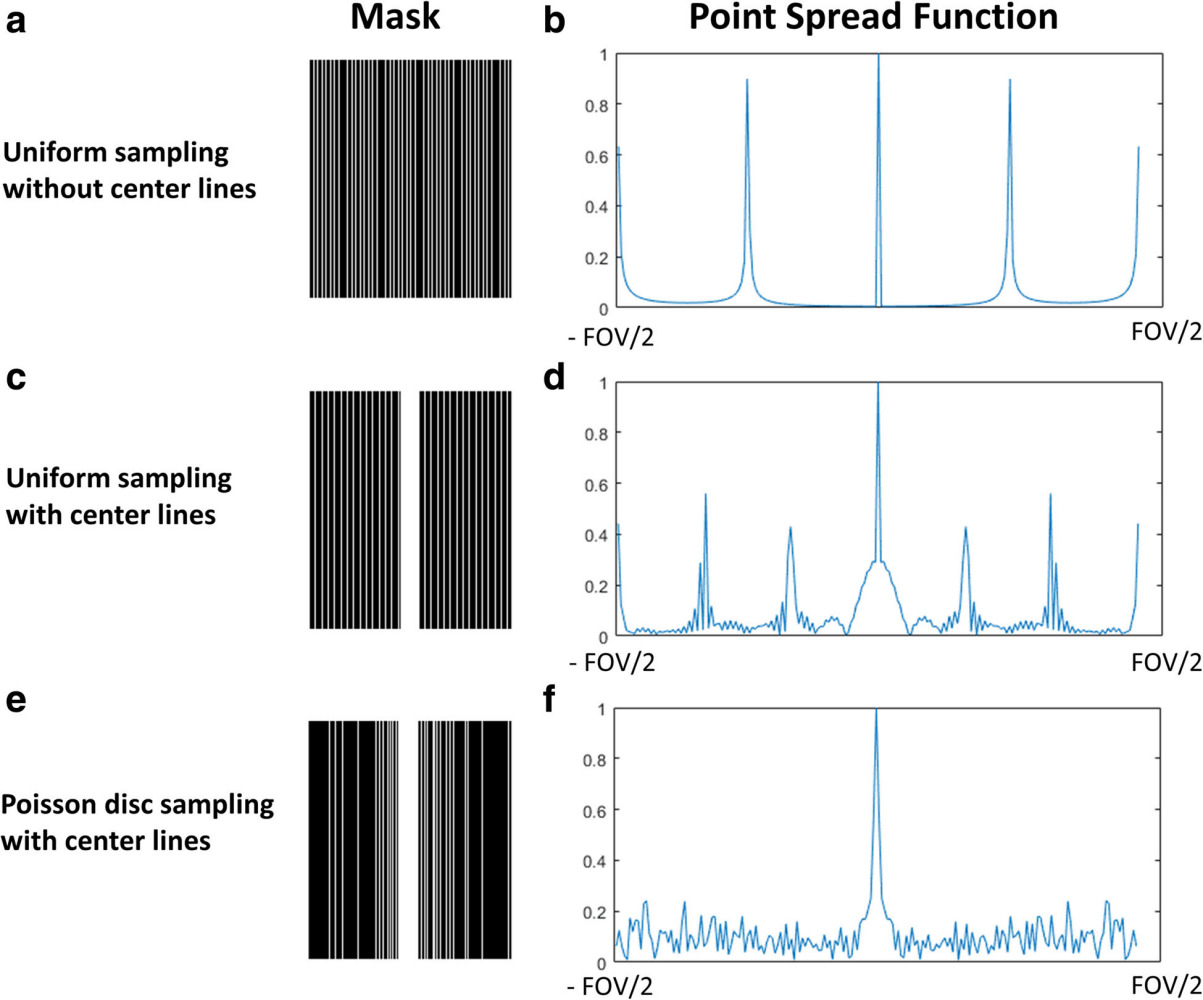
Sampling mask and point spread function comparison. Four times accelerated uniform undersampling without center lines (**a**, **b**), uniform undersampling with center lines (**c**, **d**) and Poisson disc undersampling (**e**, **f**)

Figure 2 shows the sampling mask and point spread function (PSF) of 3 different sampling patterns. A sheared grid sampling strategy (Fig. 2a-b) is usually used for k-t PCA reconstruction. In the absence of motion, the coherent aliasing is optimal for parallel imaging reconstruction since there is minimal overlap of aliased image structures. However, in the presence of respiratory motion the high-energy side lobes result in coherent residual aliasing artifacts.

In practice, the center of k-space is typically fully sampled in the k-t PCA technique to derive the temporal basis functions (Fig. 2c-d). This data can be used to reduce the coherency of aliasing when using an iterative k-t PCA approach. It results in broadening of the main lobe of the PSF in exchange for lowering the amplitude of the aliasing side lobes. However, the above 2 uniform undersampling strategies both generate high energy aliasing peaks of more than 50% of the main lobe.

A variable density Poisson disk undersampling strategy (Fig. 2e-f) is often used for compressed sensing techniques such as k-t SLR because it meets the CS criteria of incoherent sampling. By using this sampling pattern, there is a broadened main lobe with low-energy incoherent aliasing side lobes with a peak amplitude of less than 20% of the main lobe. This property provides tolerance to motion. Thus, in the presence of respiratory motion, this sampling strategy could also be beneficial for k-t PCA reconstruction.

### Image reconstruction

Following image registration as described above, the phase shifted k space data is used to perform standard k-t PCA/SENSE or k-t SLR/SENSE image reconstruction. Coil sensitivity maps are created using the temporally averaged fully-sampled k-space center through a modified Walsh method [30] and used for both reconstruction techniques.

#### K-t PCA reconstruction

For k-t PCA/SENSE algorithm, the center 10 fully sampled phase-encoding lines in k space are used as training data for principle component analysis to obtain the temporal basis functions. K-t PCA reconstruction is performed using an iterative k-t PCA approach as described previously [11].

#### K-t SLR reconstruction

The k-t SLR/SENSE algorithm is performed using iterative singular value thresholding (IST) [31]. For fair comparison to the k-t PCA technique, image sparsity was not exploited during image reconstruction.

#### Non-rigid motion correction reconstruction

To evaluate the performance of rigid vs non-rigid motion correction approach, iterative k-t PCA and k-t SLR were performed using non-rigid registration and a non-linear conjugate gradient solver. Non-rigid k-t PCA reconstruction was performed using iterative k-t PCA as described above. Non-rigid registration was performed by Advanced Normalization Tools (ANTs) using symmetric normalization including affine and deformable transformation, with mutual information as optimization metric [32]. For non-rigid k-t SLR, the low rank constraint was enforced on the non-rigid motion corrected data. These approaches were chosen to be able to directly compare k-t PCA and k-t SLR with rigid registration approaches. Images were also reconstructed using k-t FOCUSS [33, 34] which is a compressed sensing technique which uses a motion-estimation motion-correction strategy. The key-frame was derived from the temporal averaged data.

### Numerical phantom validation

In order to evaluate the performance of the different undersampling patterns (sheared-grid and Poisson-disk, both with 10 fully sampled lines at the k-space center) and reconstruction strategies (k-t PCA and k-t SLR), we utilized a CMR-XCAT phantom with realistic respiratory motion [35]. To simulate the image quality of CMR perfusion images, noise was added to the phantom. Raw data with 160 phase encoding lines were simulated.

For the sheared-grid sampling, a variable-density k-t sampling which included a 4× accelerated sheared-grid pattern and a fully sampled central 10 phase encoding lines was used. For the variable density Poisson disk sampling pattern, the central 10 phase-encoding lines were fully acquired in each frame while the outer 30 phase-encoding lines were undersampled following a variable density Poisson disc distribution in the phase encoding direction and a uniform Poisson disk sampling along the temporal direction. Images sampled with these two sampling strategies were reconstructed using k-t PCA and k-t SLR with and without MC separately.

To test the performance of this motion compensation strategy, the Poisson disc undersampled k-t PCA and k-t SLR reconstruction results with and without MC were compared. For the phantom studies, the image quality in the heart ROI was assessed in a ROI around the heart by comparison to the fully sampled images using normalized RMSE and SSIM [36]. These metrics were compared among all frames using two-way ANOVA with each image frame serving as a separate block. Post hoc comparisons between techniques were performed using a paired difference test with Tukey correction for multiple comparisons. All statistic tests were performed using SAS (version 9.4, SAS Institute Inc., Cary, NC).

### Human imaging

Resting first-pass perfusion imaging was performed in 12 subjects undergoing clinically ordered CMR studies. Written informed consents were obtained from all subjects, and imaging studies were performed under institutional review board (IRB) approved protocols. Perfusion imaging was performed using 0.075 mmol/kg Magnevist (Bayer AG, Leverkusen, Germany) injected intravenously at a rate of 4 mL/s followed by 25 mL saline flush at 4 mL/s.

CMR scanning was performed on a 1.5 T scanner (MAGNETOM Avanto, Siemens Healthineers, Erlangen, Germany) at the University of Virginia Medical Center. Multi-slice 2D saturation-recovery first-pass gadolinium-enhanced data were collected using a standard body phased-array radiofrequency (RF) coil. Sequence parameters included: FOV = 320 mm, TR = 2.4 msec, TE = 1.19 msec, saturation recovery time = 100 msec, voxel size = 2.0 × 2.0 mm^2^, matrix size = 320 × 160, slice thickness = 8 mm. For each patient, 3 short-axis slices were acquired per heartbeat for 50–70 heartbeats. We first performed prospectively rate-4 (Pro R4) accelerated first-pass perfusion data in 10 patients with 96 msec acquisition window for each slice. We also collected two data sets with prospectively rate-6 (Pro R6) accelerated data, where the acquisition window was further shortened to 64 msec per slice. This was done to maintain the same voxel size for comparison to the prospective rate 4 data.

Image quality was assessed by 2 experienced cardiologists blinded to the reconstruction technique. To facilitate blinding, the non-motion corrected datasets and non-rigid motion corrected methods were rigidly registered by deriving the displacements from a region around the heart after reconstruction and the images were cropped around the heart. Image quality was evaluated on a 5-point scale ranging from 1 (poor) to 5 (excellent). Images were also ranked in numerical order from 1st (best) to 4th (worst) not allowing for ties. In a separate analysis, k-t PCA and k-t SLR with rigid-registration were compared to k-t PCA and k-t SLR with non-rigid registration, and to k-t FOCUSS using the same grading and ranking scheme by the expert reviewers. Statistical analysis using SAS software (SAS Institute, Cary, North Carolina, USA) was performed using an ANOVA analysis with Tukey’s Studentized Range test to correct for multiple comparisons.

## Results

### Phantom experiments

**Fig. 3:**
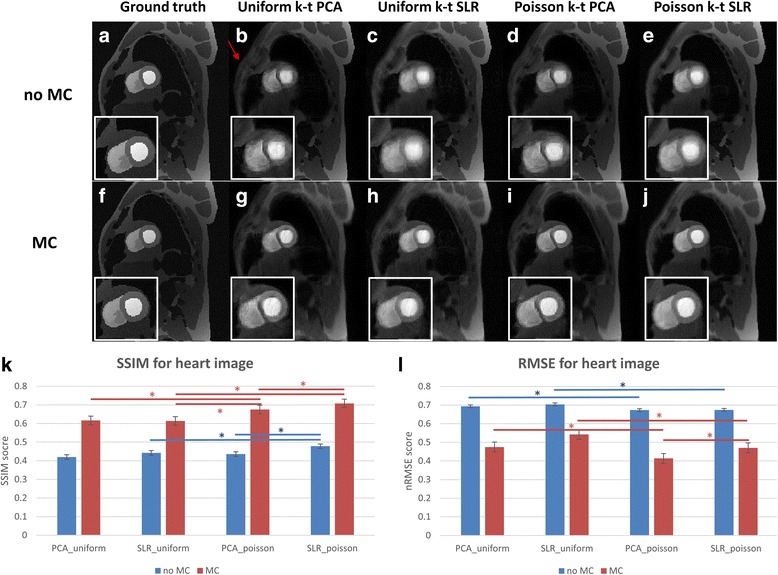
CMR-XCAT phantom of uniform and Poisson disc undersampling reconstruction with and without motion correction. The top part of the figure shows one static frame image results, and the first row corresponds to the ones without motion correction while the second row refers to the ones with motion correction. Each column represents the fully sampled ground truth (**a**, **f**), results from uniform undersampling using k-t PCA (**b**, **g**), k-t SLR (**c**, **h**), Poisson disc undersampling using k-t PCA (**d**, **i**) and k-t SLR (**e**, **j**). The red arrow indicates 1 example of the aliasing caused by undersampling. (**k**) and (**l**) are statistic SSIM and normalized RMSE scores comparing the above 4 methods with the fully sampled ground truth among all frames. * indicates *p* < 0.05. All the motion corrected results have significant higher SSIM (*p* < 0.05) and significant lower RMSE (*p* < 0.05) compared to the ones without motion compensation (not shown). CMR, cardiovascular magnetic resonance; PCA, principal component analysis; SLR, sparcity and low rank structure; SSIM, structural similarity index; XCAT, extended cardiac torso

Figure 3 shows the results from the CMR-XCAT phantom reconstructed with and without motion correction. The SNR of the phantom on this frame was 12.8. The first column in Fig. 3 shows the fully-sampled ground truth image without (a) and with (f) motion correction. In the sheared-grid sampled data reconstructed with k-t PCA (b), there are discrete aliasing artifacts resulting from incomplete decoding in the presence of respiratory motion significantly degrading image quality. As the motion strategy corrects for motion of the heart, there are still aliasing artifacts in the k-t PCA with sheared-grid undersampling (g) resulting from the chest wall which has some motion that is not corrected. For k-t PCA with Poisson disk sampling without motion correction (d) there are less discrete aliasing artifacts and image quality is slightly improved in the region of the heart. The image with motion correction (i) does not have discrete aliasing and has higher image quality. As expected, k-t SLR performs poorly for uniform undersamping without motion correction (c), resulting in some discrete aliasing, whereas for the Poisson disk sampling without motion correction (e), there are no discrete aliasing artifacts. With motion correction, k-t SLR with uniform undersampling (h) again performs poorly due to the sampling requirements of CS which are not met, whereas the Poisson disk k-t SLR (j) results have good image quality without blurring. For both k-t PCA and k-t SLR, Poisson disk sampled images have higher SSIM and lower RMSE compared to uniform sampled ones (*p* < 0.05). Moreover, for the Poisson disk sampling strategy k-t SLR has a higher similarity index than k-t PCA (*p* < 0.05). Without motion correction, there is significant spatial blurring and there are artifacts in the region of the myocardium which has lower SSIM (*p* < 0.05) and higher RMSE (*p* < 0.05).

By comparing the images in Fig. 3, the images using Poisson disk undersampling have better image quality than those using uniform undersampling. K-t SLR typically out-performed k-t PCA in the setting of respiratory motion.

### Human studies

**Fig. 4:**
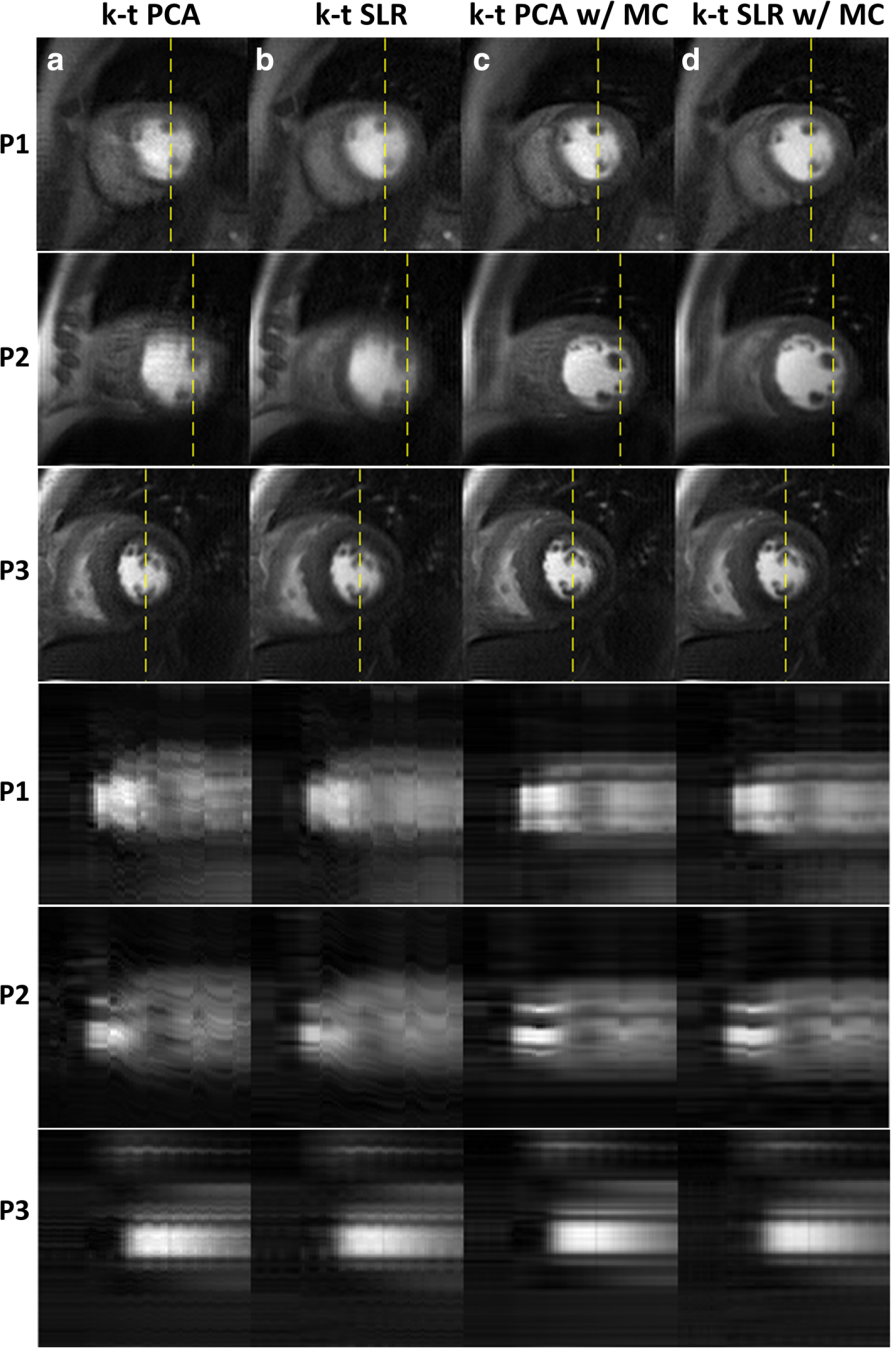
k-t PCA and k-t SLR reconstructions with and without motion compensation for patients. Comparisons of the reconstructed images (top 3 rows) and x-t profiles (bottom 3 rows) are made for reconstruction without motion correction k-t PCA (column **a**) and k-t SLR (column **b**), with motion correction k-t PCA (column **c**) and k-t SLR (column **d**). The x-t profile corresponds to the location of the dashed lines in the reconstructed images

Figure 4 shows a comparison between k-t PCA and k-t SLR reconstruction results with and without motion correction acquired with a 4× accelerated Poisson disk undersampling strategy as described above. For subjects 1 and 2, when there is a significant amount of respiratory motion, the images reconstructed with k-t PCA and k-t SLR without motion compensation have significant blurring artifacts over the heart as compared to those with motion compensation. X-t profiles of motion corrected data show sharper borders between the blood pool and the myocardium, and the trabeculations can be well visualized. For the third subject in the figure there is minimal respiratory motion. Images with motion compensation look slightly sharper than the non-motion corrected images, but the differences are subtle. Similarly, the x-t profiles are similar between the motion corrected and non-motion corrected reconstructions. Note in this figure for comparison the x-t profiles for the non-motion corrected techniques have been registered after reconstruction for display purposes.

**Fig. 5:**
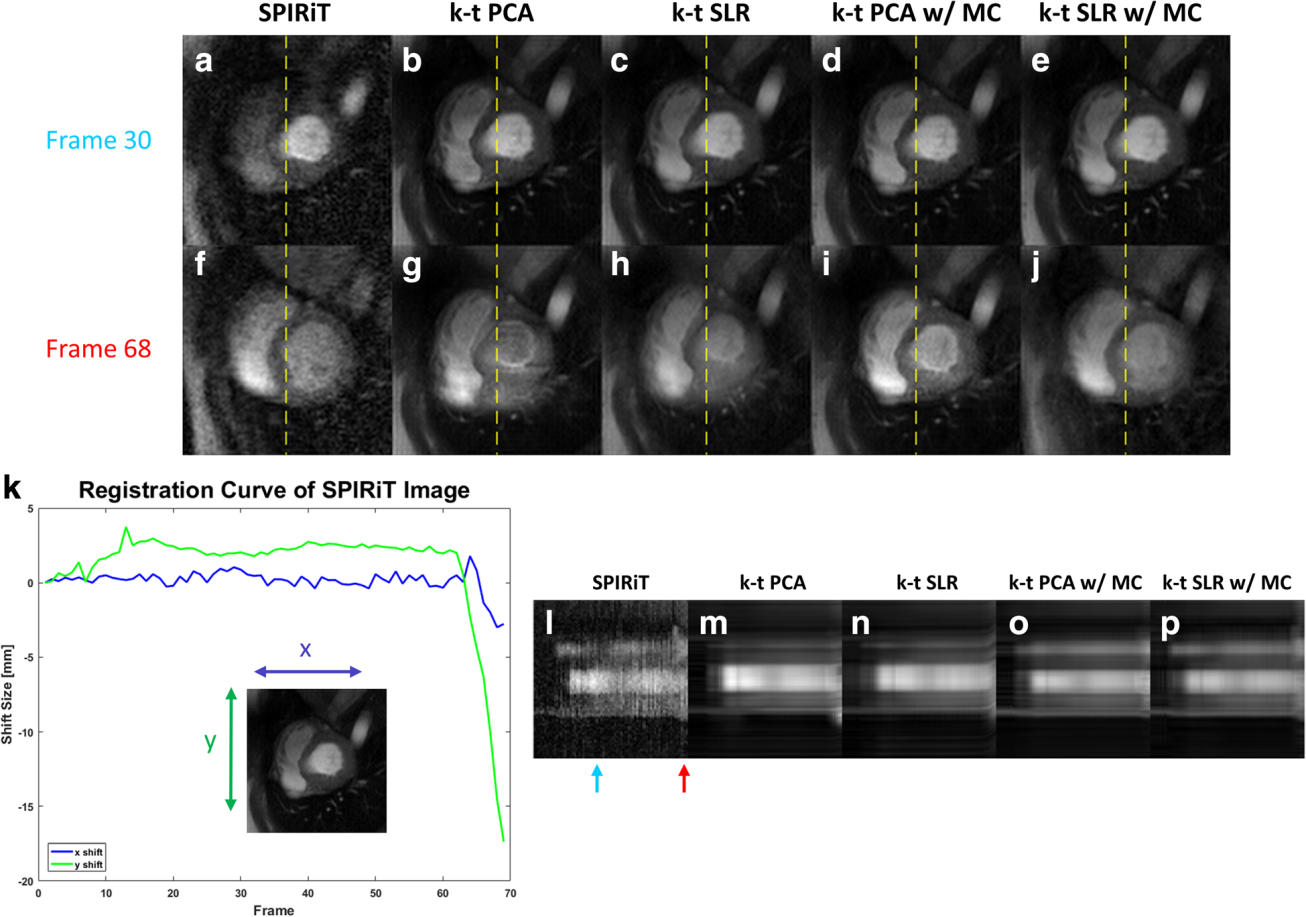
Reconstruction comparison of a subject with 6 times accelerated Poisson disc undersampling. Reconstruction results are shown at 30th and 68th frame. Each column corresponds to direct SPIRiT reconstruction with registration (**a**, **f**), non-motion corrected k-t PCA (**b**, **g**) and k-t SLR (**c**, **h**), motion corrected k-t PCA (**d**, **i**) and k-t SLR (**e**, **j**). The temporal x and y-shifts of the heart position derived from rigid registration of the SPIRIT images (**k**) demonstrate that the patient took a deep breath near frame 60. The x-t profiles for the above 5 reconstruction methods are shown (**l**-**p**) with colored arrows denoting the 30th and 68th frames

Figure 5 shows perfusion images from the 30th frame (a-e) and the 68th frame (f-j) of a first-pass dataset acquired with 6× Poisson-disk undersampling. Figure 5k shows the temporal x and y-shifts of the heart position derived from rigid registration of the SPIRiT images. The x-t profiles for the above 5 reconstruction methods are shown (l-p) with colored arrows denoting the 30th and 68th frames. In this case, the subject held his breath reasonably well until frame 65 where he took a deep breath resulting in through-plane motion. This can be clearly seen in the SPIRiT reconstructed images. Note all reconstructions demonstrate good image quality for the 30th frame. However, for the 68th frame the non-motion corrected k-t PCA and k-t SLR images have significant blurring artifacts. The motion-corrected k-t PCA images fail to show the through-plane motion. The motion-corrected k-t SLR images are able to capture this through-plane motion, albeit with some blurring of the inferior wall.

**Fig. 6:**
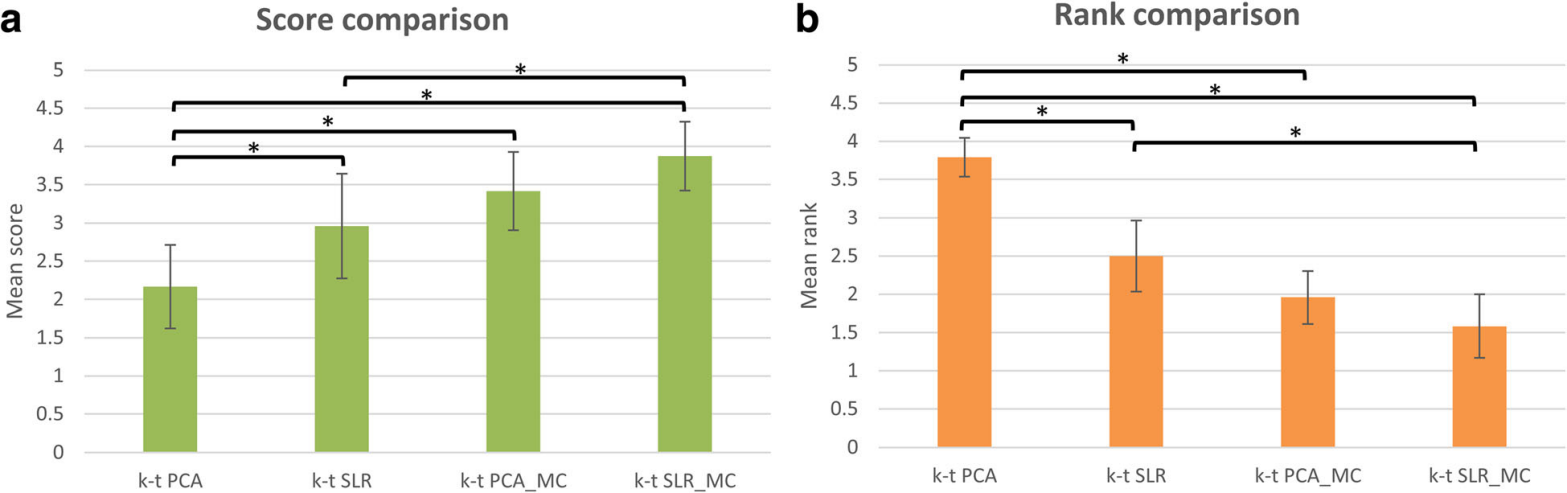
Score (**a**) and rank (**b**) comparisons of k-t PCA and k-t SLR reconstruction results with and without motion compensation by 2 experienced cardiologists. Four bars in each plot from left to right correspond to k-t PCA and k-t SLR without motion compensation, k-t PCA and k-t SLR with motion compensation. The scale of the scores range from 1 (very poor) to 5 (very good) and the scale of the ranks range from 1st to 4th. Error bars indicate the standard deviation, and * indicates significance at *p* < 0.05

Figure 6 shows the comparison of scores and ranks for the cardiologists for k-t PCA and k-t SLR reconstructions for the 10 cases acquired with 4× undersampling with and without motion-compensated reconstruction. In the score plot (Fig. 6a), the non-motion corrected reconstructions had significantly worse image quality scores than the motion corrected reconstructions (*p* < 0.05) for k-t PCA and k-t SLR. For non-motion-compensated reconstructions, there was significant higher image quality score for k-t SLR as compared to k-t PCA. Similar results were seen in the plot of the rank data. K-t PCA and k-t SLR images with motion corrected reconstructions had significant better ranks than those without motion-compensated reconstruction (*p* < 0.05). Again, there were significant differences in rank between non-motion corrected k-t PCA and k-t SLR. Although the difference between motion-corrected k-t PCA and k-t SLR is not significant, k-t SLR with motion correction had the best point estimates for mean score/rank of the techniques.

**Fig. 7:**
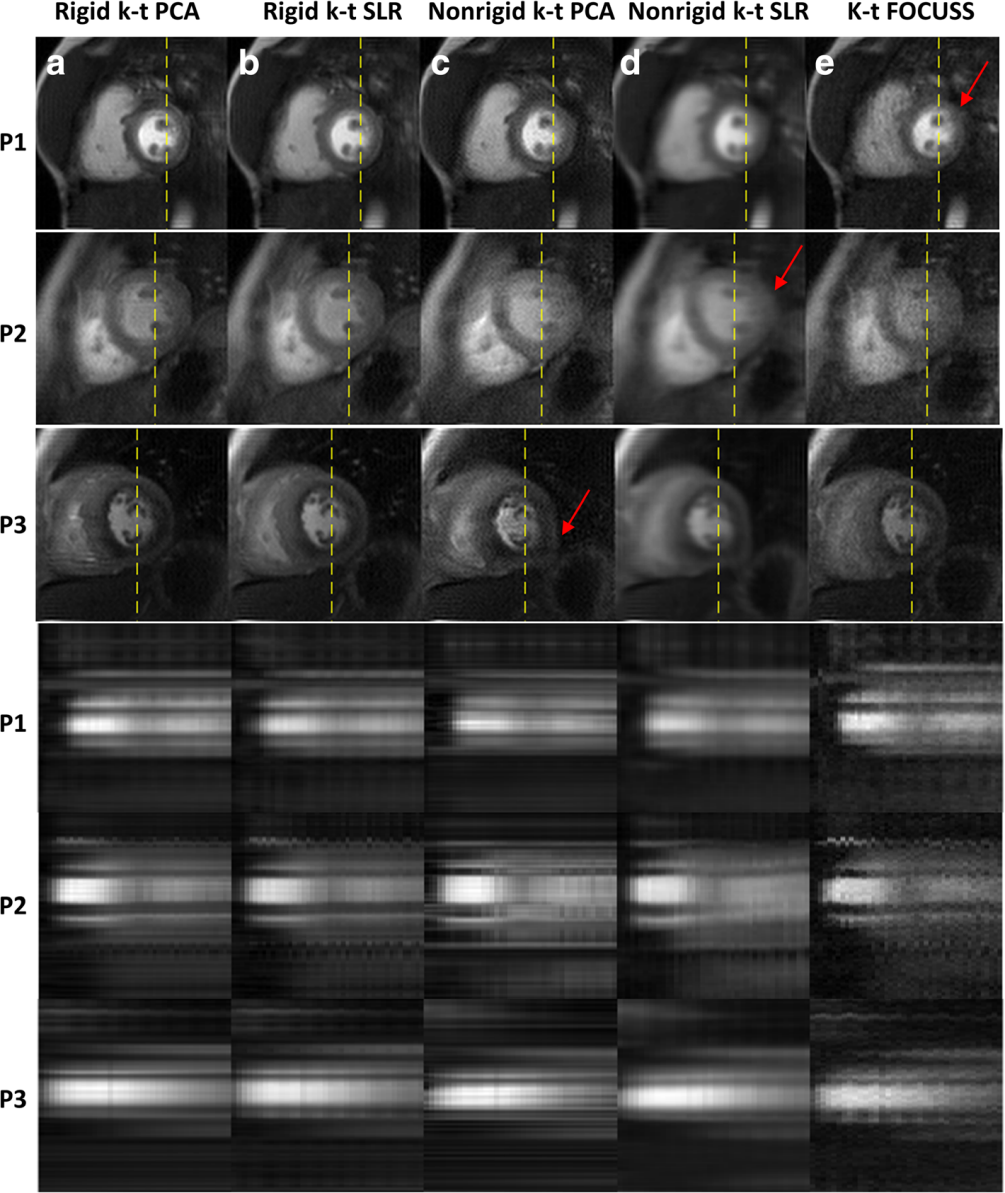
Motion correction with rigid k-t PCA/SLR, nonrigid k-t PCA/SLR and k-t FOCUSS image reconstruction results for patients. The top 3 rows are the reconstructed images at one frame and the bottom 3 rows are the x-t profiles for the dashed line pointed in the frames. Comparisons are made among the proposed motion corrected rigid k-t PCA (column **a**), rigid k-t SLR (column **b**), as well as nonrigid k-t PCA (column **c**), nonrigid k-t SLR (column **d**) and k-t FOCUSS (column **e**). Red arrows indicate distortion and blurring artifacts

**Fig. 8:**
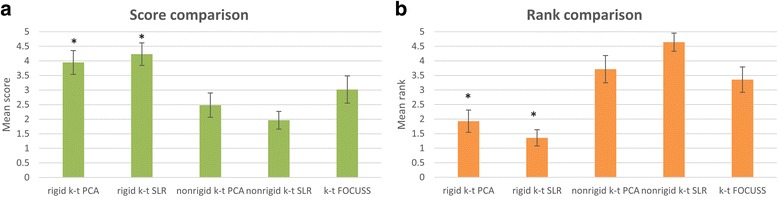
Score (**a**) and rank (**b**) comparisons among motion correction rigid k-t PCA/SLR, nonrigid k-t PCA/SLR and k-t FOCUSS reconstruction results by 2 experienced cardiologists. Five bars in each plot from left to right corresponds to motion corrected rigid k-t PCA and k-t SLR, motion corrected nonrigid k-t PCA and k-t SLR, and motion corrected k-t FOCUSS. The scale of the scores range from 1 (very poor) to 5 (very good) and the scale of the ranks range from 1st to 5th. Error bars indicate the standard deviation, and * indicate the significance between rigid k-t PCA/k-t SLR and other methods

Figure 7 shows the results for the comparison of rigid-motion corrected k-t PCA and k-t SLR, non-rigid corrected k-t PCA and k-t SLR and k-t FOCUSS. As compared to our rigid-registration approach, the non-rigid registration correction using k-t PCA and k-t SLR tended to have geometric distortion and blurring. K-t FOCUSS had a lower apparent SNR and also had some blurring and aliasing artifacts in frames with significant motion. The x-t profiles in the bottom rows show more temporal blurring and noise as compared to our proposed approach. As shown in Fig. 8, the image quality scores and ranks were significantly higher (*p* < 0.05) for k-t PCA and k-t SLR with rigid motion correction as compared to the other motion-corrected reconstruction approaches.

## Discussion

In this work a simple robust motion correction strategy for the reconstruction of undersampled motion corrupted dynamic perfusion CMR data has been proposed. The method is based on deriving rigid translations of a ROI around the heart, and applying the linear phase shifts to the whole raw k-space as a pre-processing step. Our reconstruction strategy provides a simple way to remove respiratory motion artifacts in the heart region at the expense of some motion degradation of anatomy remote from the heart.

In this way, motion is corrected in the vicinity of the heart resulting in improved k-t accelerated and CS reconstruction of the heart. This property makes the strategy suitable when only a region around the heart is of interest, such as during first-pass perfusion. Because of the large contrast change in the ventricular cavities during first-pass perfusion imaging, the automatic detection of the heart ROI based on the temporal standard deviation of signal intensity performs well for this application. The heart ROI was successfully detected in all cases. This technique was evaluated both in a CMR-XCAT phantom and in patients undergoing clinical perfusion studies. The strategy significantly reduces respiratory motion artifacts from poor breath-holding, thus improving image quality in the setting of respiratory motion.

While a sheared-grid sampling pattern should theoretically be optimal in terms of separating aliased replicates in x-f space [10], in the setting of respiratory motion a prior perfusion study using k-t SENSE has demonstrated significant discrete aliasing artifacts which can significantly impact image quality [20]. We showed a similar result in the CMR-XCAT phantom with a sheared grid pattern, but demonstrated that image quality in the setting of respiratory motion is improved by using a Poisson disk sampling pattern which has a less discrete aliasing pattern. In the patient studies, we also demonstrated that using rigid motion correction around the heart further improves the quality of image reconstruction for k-t PCA in the setting of significant respiratory motion.

For k-t SLR, an example of a compressed-sensing reconstruction which may be sensitive to respiratory motion, it is demonstrated significant improvement in image quality using this rigid motion correction approach in both the CMR-XCAT phantom and patient studies. In comparison of the techniques (k-t PCA and k-t SLR) both with and without motion correction, we found the best results from motion-corrected k-t SLR. This is particularly demonstrated in the case shown in Fig. 5, where the patient has obvious through-plane motion resulting in significant shape changes of the heart during a small number of temporal frames at the end of the acquisition. As k-t PCA models the temporal basis functions directly from the training data, when motion occurs over a small number of frames, the k-t PCA calibration may become biased towards the motion-free frames and may not accurately capture changes due to through-plane motion. In k-t SLR, the low-rank constraint is enforced iteratively with soft-thresholding, potentially reducing sensitivity to bias from a small number of discordant frames.

While currently more complex techniques for motion corrected k-t acceleration exist [21, 24, 37, 38], their performance is highly dependent on non-rigid registration, which is sensitive to image quality related factors. As we had tested in Fig. 7, in the nonrigid registration cases, the complex computation has a potential to results in geometric distortion of the images and blurring. In addition, running nonrigid registration motion correction methods require longer image reconstruction time, limiting their general applications. By comparison, the approach utilized in this manuscript has a number of advantages. Firstly, rigid translations are easily determined even in images with some degree of residual spatial aliasing where occasionally non-rigid registration will fail or attempt to register the aliasing artifacts. Secondly, as only translations are being estimated, there is no possibility of the registration causing geometric distortions of the heart that can occur with non-rigid registration techniques. Considering that the registration only phase shifts the k-space data, it avoids spatial blurring resulting from the repeated application of non-rigid registration operators during conventional iterative motion-compensated reconstruction [39]. Furthermore, this registration step could be easily added as a pre-processing step for current k-t PCA and k-t SLR pipelines. Although we did not evaluate the technique during stress perfusion in this study, we would expect to see similar findings during stress perfusion acquisition.

This reconstruction technique has some inherent limitations. As with most 2D registration techniques, the registration is sensitive to through-plane motion. While non-rigid motion techniques can register the heart on beats with mistriggering, rigid registration does not allow any deformation of the heart resulting in some residual motion of the heart. In this study, we sought to use this technique to correct for poor breath-holding rather than performing free breathing. However, as these were all clinical patients, the majority of patients had difficulty holding their breath for 50–70 heart beats which is about 30–60 s. The proposed technique may not work if the patient takes very large breaths resulting in significant through-plane motion.

## Conclusion

A simple robust rigid motion compensation strategy is presented for dynamic 2D cardiac perfusion CMR. It greatly reduces motion artifacts and improves image quality for the standard k-t acceleration (k-t PCA) and CS (k-t SLR) techniques in setting of respiratory motion.

## Abbreviations

ANT: Advanced normalization tools
BLAST: Broad-use linear acquisition speed-up technique
CAD: Coronary artery disease
CMR: Cardiovascular magnetic resonance
CNR: Contrast-to-noise ratio
CS: Compressed sensing
ECG: Electrocardiogram
FOV: Field-of-view
GRAPPA: Generalized autocalibrating partially parallel acquisition
MC: Motion compensation
PCA: Principal component analysis
PSF: Point spread function
RF: Radiofrequency
RMSE: Root mean square error
ROI: Region-of-interest
SENSE: Sensitivity encoding
SLR: Sparcity and low rank structure
SNR: Signal-to-noise ratio
SPIRiT: Self-consistent parallel imaging reconstruction
SSIM: Structural similarity index
TE: Echo time
TR: Repetition time
XCAT: Extended cardiac torso digital
