# Correction to: Simple motion correction strategy reduces respiratory-induced motion artifacts for k-t accelerated and compressed-sensing cardiovascular magnetic resonance perfusion imaging

**DOI:** 10.1186/s12968-018-0439-x

**Published:** 2018-03-26

**Authors:** Ruixi Zhou, Wei Huang, Yang Yang, Xiao Chen, Daniel S. Weller, Christopher M. Kramer, Sebastian Kozerke, Michael Salerno

**Affiliations:** 1 Department of Medicine, University of Virginia Health System, Charlottesville, VA USA; 2 Department of Biomedical Engineering, University of Virginia Health System, Charlottesville, VA USA; 3 Medical Imaging Technologies, Siemens Healthineers, Princeton, NJ USA; 4 Department of Electrical and Computer Engineering, University of Virginia, Charlottesville, VA USA; 5 Department of Radiology and Medical Imaging, University of Virginia Health System, Charlottesville, VA USA; 6 Department of Information Technology and Electrical Engineering, Institute for Biomedical Engineering, University and ETH Zurich, Zurich, Switzerland

## Correction

Figure 1 of this original publication [1] contained a minor error as one of the lines in the “Reconstruction pipline” was not visible. The updated Fig. 1 is published in this correction article.

**Fig. 1:**
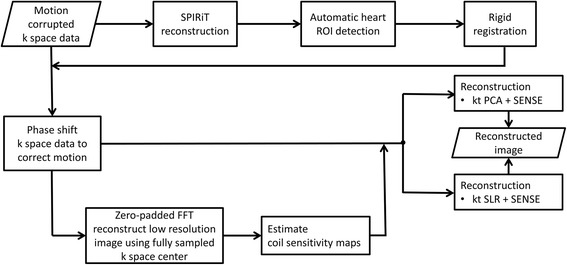
Corrected Reconstruction pipeline
